# Clothing Pressure Alters Brain Wave Activity in the Occipital and Parietal Lobes

**DOI:** 10.1515/tnsci-2019-0013

**Published:** 2019-04-23

**Authors:** Yunjuan Liu, Yan Wang

**Affiliations:** 1 Collaborative Innovation Center of Modern Clothing Technology Minjiang University, Fuzhou, China; 2 The Department Electrical and Computer Engineering, The University of Texas at San Antonio, San Antonio, USA

**Keywords:** clothing, electroencephalogram, EEG source analysis, psychology of fashion

## Abstract

Despite the importance of clothing pressure discomfort in the undergarment industry, a reliable unbiased measurement of clothing pressure discomfort has not been well-established. In the present study, we investigate changes in brain wave activity as a potential objective and consistent measuring tool for clothing pressure discomfort. We recorded α wave activity in 5 functional regions (30 channels) of the brain in 10 females with or without a girdle. We determined that α wave power spectrum significantly increases when the girdle is worn compared to when it is not worn, specifically in the parietal and occipital regions. These findings suggest that clothing pressure exerted by wearing a girdle mostly stimulates the parietal and occipital regions and that these regions should be investigated in future studies using α wave energy to measure clothing pressure discomfort.

## 1 Introduction

Girdles are functional undergarments that fit tightly around the waist to improve waist and abdomen shape. The excessive pressure of overly tight girdles not only result in discomfort but also squeeze the organs within the body, resulting in organ and neural system disorders, poor blood circulation, menoxenia, poor digestion and constipation[1, 2, 3, 4]. Thus, evaluation of clothing pressure comfort is one of a key technique in the underwear industry.

Traditionally, clothing pressure comfort is evaluated subjectively. However, this evaluation method results in unstable and unreliable data. To reduce variability, novel evaluations systems incorporate principles of physio-psychology theories and physical discomfort.

One such evaluation method utilizes brain waves. Brain wave-based evaluation has recently been used to objectively assess clothing aesthetics [5, 6] and heat, moisture [7], touch [8], and pressure comfort [9]. While these studies [10, 11] detected a change in the ratio of time a specific brain wave, the α wave, was dominant, these ratios (α wave indexes) varied considerably and were considered unreliable. Thus a more consistent measure of changes in the α wave needs to be developed in order for brain waves to be used as a more objective measure for clothing pressure comfort.

In the present study [12, 13] we examined α wav intensity in various regions of the brain stimulated by different clothing pressures along the waist to determine their utility as an alternative measurement of clothing pressure discomfort. Our work lays the foundation for relevant research on objective clothing pressure measurements and contributes to the physio-psychology study on the body’s response to clothing pressure stimulus.

## 2 Experiment

### 2.1 Subjects

A total of 10 young females with similar body shapes were chosen as the subject, and the feature data of their body shapes should fall within the pre-defined range. In order to ensure consistency with subsequent brain wave experiment, the subjects must be as healthy as required in the brain wave test. The range of the physical characteristics is shown in Table 1.

**Table 1:** Range of physical characteristics for experimental subjects

Age	Height	Weight	BMI	Under Bust	Waist Circumference	Abdominal Circumference	Hip Circumference
24±2	160±4	49±3	19-22	72±3	64±3	72±3	87±3
(Year-old)	(cm)	(kg)	(kg·m^2^)	(cm)	(cm)	(cm)	(cm)

### 2.2 Experimental material

**Figure 1:**
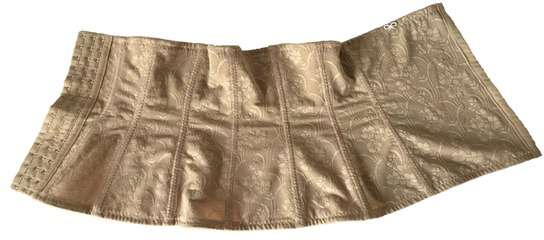
The girdle in experiment

A girdle (Figure 1) consisting of nylon and spandex was utilized in the study (Table 2). The girdle was designed to provide common girdle functions including breast support, waist narrowing, abdomen flattening and hourglass body curvature. Girdles size S (waist: 61-67cm; circumference 83-93) was used (Table 2).

**Table 2:** Information of experimental samples

Fabric	Fiber	Quantity of Plastic Bone	Position of Hook	Size	Applicable Size Range for No. 64
Elastic Mesh Fabric	Fabric: Nylon 81%, Spandex 19%	7 PCS	Frontal	64 (S)	Waist Circumference: 61-67cm
	Lining: Nylon 74%, Spandex 26%				Hip Circumference: 83-93cm

The girdle consists of a two-layer elastic mesh distributed bilaterally, two small non-elastic sections located along the waist with one larger elastic component, an piping, and a layer of non-elastic fabric in the middle of the two layers of elastic cloth. The girdle has hooks on both sides of the non-elastic part. The piping acts as a support and separates the fabric in the various girdle sections to produce a smooth curved appearance along the waist.

### 2.3 Experimental equipment

**Figure 2:**
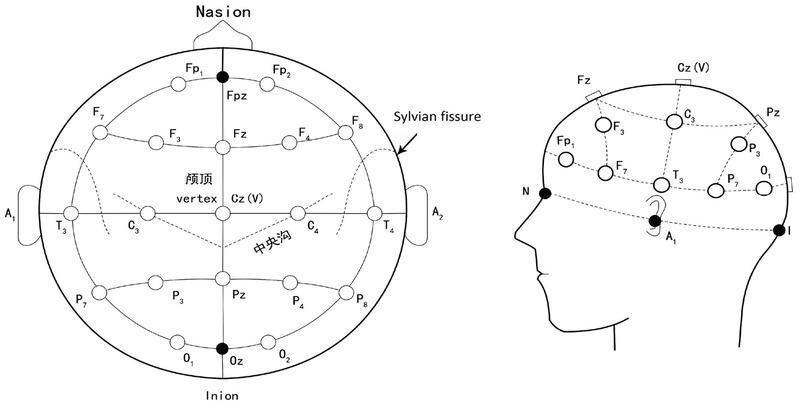
Measurement placement

The U.S. Neuroscan EEG/ERPS NuAmps, portable 40-electrode digital DC, and EEG/ERPs amplifier were used to record brain waves. Five

functional regions of the brain were defined: the frontal lobe region F (FP1, FP2, F3, F4, FZ, F7, F8, FT7, FC3, FCZ, FC4, FT8), lobes temporalis region T (T3, T4, TP7, TP8, P7, P8), central region C (C3, CZ, C4), parietal region (P3, PZ, P4, CP3, CPZ, CP4), and the occipital region O (O1, O2, OZ). The electrodes were organized along these regions as shown in Figure 2.

### 2.4 Experimental conditions

Data collection was conducted in the brain wave laboratory at a constant temperature （25±2℃) and moisture (65±2℃). The laboratory was effectively shielded from electromagnetic waves. Participants had sufficient sleep the night before testing, and cleaned their hair, did not apply oil or fat substances, and did not perform violent exercises or undergo emotional fluctuations prior to testing. During the experiment, the participants wore the girdle (without a bra) in order to eliminate interference from other clothes.

### 2.5 Experimental process

The process of brain wave measurement is shown in Table 3. Brainwaves for participants were recorded with and without the girdle with the electrode set at different positions. Prior to each recording session, the participants were allowed to relax by sitting quietly and listening to light music for 30 minutes. Brainwaves were then recorded for 1 minute. The subjects were not permitted to do anything irrelevant to the experiment. Prior to recording sessions, the electrode cat was connected to the amplifier and specific parameters were set.

**Table 3:** Measurement process

Wear Girdle		Not Wear Girdle	
Relax	Brainwave Reading	Relax	Brainwave Reading
	Sitting		Sitting
30min	1min	30min	1min

During data recording, the electric resistance was maintained at a level under 5 KW and the digital sampling rate was 1000Hz. The average brainwave of the electrodes when they are placed above the mastoid bone was used as a reference brainwave. A set of brain wave data was collected beforehand to determine that the baseline and electrodes were correctly set up.

### 2.6 Data processing and analysis

Power spectrum analysis was adopted for frequency-domain processing of the brain wave data. Relative brainwave spectrum was used as a metric of brain wave rhythm in order to compare brainwave spectrums across different individuals. The relative power is defined as the ratio between the absolute power of a specific frequency band and the total power of all frequency bands.

The brain wave data from 30 electrodes was pre-processed by implementing the following steps: electrooculographic integration, DC correction, electrooculographic elimination, baseline correction, filtering, MARK-free brain wave fragmentation (511ms-long brain wave signal with 512 sampling points), and frequency-domain overlapped averaging (Hanning window function). The pre-processed data then underwent offline analysis to generate brain wave power data across four frequency bands. After which, SPSS 15.0 was utilized to analyze the relative power of the a wave under each condition.

## 3 Results

### 3.1 Impact of clothing pressure on the a wave power

To determine whether α wave power can be used as a reliable measure for clothing pressure comfort, we examined changes in power in 10 female participants with and without a girdle using an electrode located along the parietal region (point P3). Data from subject B is shown to illustrate data analysis results.

**Figure 3:**
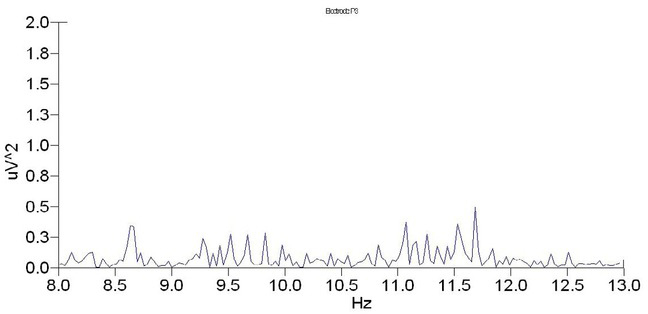
Power spectrum of subject B with the girdle

**Figure 4:**
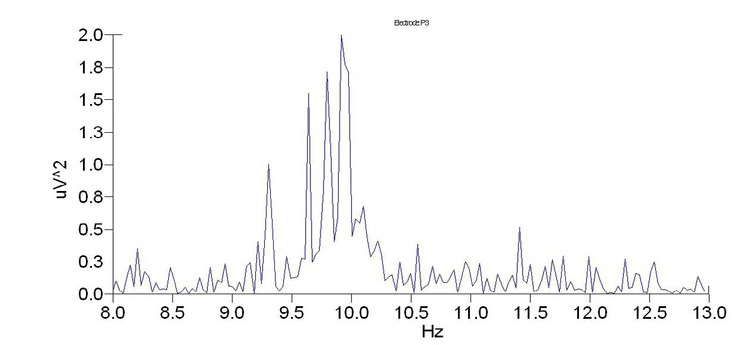
Power spectrum of subject B without the girdle

As shown for subject B, the maximum power spectrum of the α wave significantly decreases when pressure is applied to the waist (Figure 3; 0.6mV2) when compared to when no pressure is applied (Figure 4; 2mV2), indicating that the a wave intensity decreases when pressure is applied to the waist.

Energy analysis of the a wave power spectrum revealed that wave length intensity was significantly different for participants when the girdle was worn when compared to when the girdle was off (Table 4; P≤0.01). Taken together, the data suggests that α wave intensity significantly differs based on clothing pressure.

**Table 4:** Analysis on intensity of a wave with and without girdle

Factor	III type sum of squares	df	Mean square	F	Sig.
Position	23.997	29	.827	18.246	.000
Status	3.564	1	3.564	78.584	.000
Position×Status	1.392	29	.048	1.058	.381

### 3.2 Impact of clothing pressure on a wave power under different electrode locations

**Figure 5:**
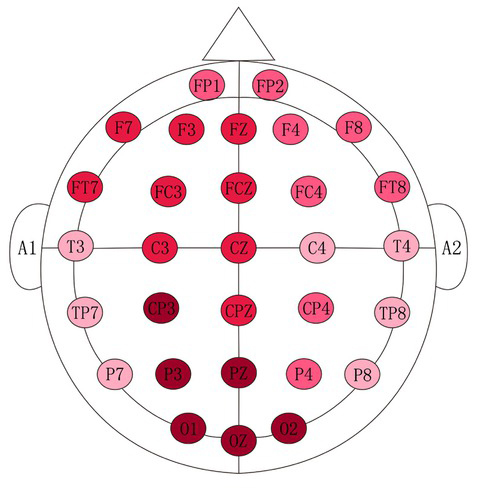
a wave power spectrum of 30 electrodes distribution map

To determine if changes in α wave intensity are impacted by the location of the electrode, we compared α wave intensity recorded at 30 different locations when participants were and were not wearing a girdle. Duncan analysis of

homogeneous subsets, where the electrode location was defined as a subgroup, revealed that the 30 tested electrode locations could be grouped into four subsets: subset 1 in the occipital and parietal regions (CP3, PZ, P3, O1, O2, OZ), subset 2 in the frontal, central and parietal regions (FC3, FZ, F7, FT7 , FCZ, CPZ, C3, F3, CZ), subset 3 in the frontal and parietal regions (FT8, FC4, CP4 , FP2, F4, P4, FP1, F8), and subset 4 in the temporal region (C4, P8, T4, TP8, T3, TP7, P7) with the exception of location C4 located in the central region. These findings indicate that the a wave power spectrum recorded from electrodes in locations within the same subset were similar. In Figure 5 using different red to show different wave power, the darker color means the higher a wave power. This figure clearly indicates that clothing pressure exerted by wearing a girdle mostly stimulates the parietal and occipital regions.

### 3.3 Individual difference between subjects

To determine if electrode locations are similar among individuals, the results of the Duncan analysis of homogeneous subsets for each participant was compared. Data analysis showed that the grouping of locations with similar differences in α wave intensities for clothing state (wearing or not wearing girdle) differs for each individual. For example, Duncan homogenous subset analysis of brain wave data recorded from subject A revealed that the 30 electrode locations could be classified into 6

subsets: subset 1 in the occipital region （CP3, CPZ, CP4 , O1, O2, OZ）, subset 2 in the parietal and frontal regions （P3, PZ, P4, F3, FZ, F4), subset 3 along the frontal region （FP1, FP2, F7, F8, FT7）, subset 4 in the frontal lobe region （FC3, FCZ, FC4, FT8）, subset 5 along the central region （T3, C4, C3, CZ, TP7）and subset 6 within the temporal region. （T4, TP8, P7, P8）with some exceptions (T3 of subset 5 was located in the temporal region). However, in both subjects A and B, electrodes recording similar α wave intensities were located in close proximity to each other. In order of descending differences in a wave spectral energy between wearing and not wearing a girdle, the subset recording location options for the subsets of electrode locations were the occipital, parietal, frontal, central, and temporal regions.

## 4 Discussion

The present study demonstrated that brainwaves display clearly different patterns under different clothing pressures. Participants wearing girdles were found to have significantly lower a wave spectral energy than when the girdle was not worn, indicating that increased clothing pressure inhibits the a wave.

Our findings are consistent with Yin [10] study investigating the length of time that the α wave is the dominant brain wave during different clothing pressures. They determined that the α wave index (the ratio of time that the α wave was dominant to the total time clothing pressure was experienced) decreased with increasing clothing pressure

The presented findings and evidence from earlier studies suggest that the α wave may change during uncomfortable experiences. This hypothesis is bolstered by Sisode [14], Nie [15] et al. studies on the relationship between a wave intensity and emotional and mental states.

In the study, α wave intensity was high in the occipitalia and pars cupularis regions of the brain of relaxed participants. However, when the participants experienced anger, fear or excitement, a wave intensity decreased. The decrease in α wave intensity during emotional extremes and during increased clothing

pressure, suggests that decreases in α wave intensity may indicate feelings of discomfort or uneasiness.

We also determined that changes in α wave power differs in different regions of the brain and that similar changes in power occur in regions in close proximity to each other. Duncan homogenous subset analysis of the difference in a wave power spectrum at 30 electrode locations showed that the subset distribution is consistent with the functional regions of brain. The change in α wave spectral energy at different clothing pressures was greatest in parietal and occipital regions. No significant changes were observed in the frontal and temporal regions. Hence, clothing pressure primarily stimulates the occipital and parietal lobes of the brain.

These significant changes in the parietal and occipital regions may be due to the fact that the α wave is highest in the occipitalia and pars cupularis, located in the occipital lobe [16]. The parietal and occipital lobes are also major brain regions activated by somatosensory stimulus. The α wave is highest in the occipital lobe, followed by the parietal, frontal and temporal lobes [17].

Future studies on whether the stimulated brain regions vary when different body parts are under clothing pressure would further elucidate the relationship between changes in α waves and clothing pressure sensations and to develop a more refined and objective measurement of clothing pressure comfort for the real-world applications.

## 5 Conclusions

(1) a wave power decreases under increased clothing pressure, demonstrating that clothing pressure on the waist inhibits a wave energy, indicating increased discomfort.
(2) The difference in a wave spectral energy under different clothing pressures were significant in occipital and parietal regions, but not in frontal, central and temporal regions of the brain. These findings suggest that the occipital and parietal regions constitute the major brain areas stimulated by clothing pressure and would provide optimal locations for recording changes in α wave energy in future studies.
